# New Elements of Operative Surgery

**Published:** 1857-01

**Authors:** 


Art. V.—*New Elements of Operative Surgery*. By Alf. A. L. M. Velpeau. Fourth edition, with additions, by George C. Blackman, M.D., Professor of Surgery in the Medical College of Ohio, &c. &c. New York. S. S. & W. Wood. 1856.

If there is anything in a book which we dislike more than another it is a long title-page. For this reason, we refrain from copying that of Mons. Velpeau's treatise; which, with the names and appendages of the author, translator, annotator, and editor, exhibits a most formidable array, and is almost long enough to reach, in a straight line, from our sanctum to the Rocky Mountains. We learn from this cumbersome page that the present is the fourth American edition, the first having been issued in 1844; a circumstance showing a just appreciation, on the part of the profession, of the value of this great work. We are, however, not quite sure that this edition is, legitimately, anything more than the second; for we are strongly inclined to believe, from a careful examination of the first three issues, that they were precisely identical, in matter, paper, and typography, or, what is the same thing, that they all wore precisely the same features, and the same dress. The truth is, the work was—what a scientific work never should be—originally stereotyped, and hence, as one imprint, probably not a large one, became exhausted, another was immediately issued in the same manner, without a jot or tittle of alteration, amendment, or improvement. We do not complain of such a proceeding; it may be all right, morally and scientifically considered; still it does not, we confess, strike us in this light, but rather as a *trick*, designed to create the impression that a work so treated, enjoys an immense degree of popularity, when, in fact, the very reverse may be true. We might refer, if we felt so inclined, to an instance where a work of very questionable popularity, published not a thousand miles off, has, in less than three years, been thus passed through four editions, not one of which, probably, exceeded two hundred and fifty copies. Now, when a work *behaves* in this way, it sails under false colors, and is unworthy of the society of respectable books. A publisher, finding that he has made a bad bargain, might possibly be excusable for playing such a game, but no author, who has any pretensions to morality, should be guilty of any connivance of the kind. The former has purchased the work as a commercial speculation, and it is his duty to make as much capital out of it as he can. But it is very different with an author. If his work is a failure, he loses only his time, and ought to be satisfied that he fares no worse. If his work is stereotyped, it is easy enough for him to strike off, every few months, a hundred or two hundred copies, and to palm them off upon the profession as a new edition; and, consequently, as an evidence of the rapid sale, and great popularity of his production. All medical works, published in this country, whether systems or monographs, have an average edition of from twelve hundred to two thousand copies; and hence the publisher has always reason to congratulate himself, if he can dispose of it, in two or three years from the time it leaves the press. There may, occasionally, be an exception; but this only proves the rule, and we are quite sure that this was not the case in the instance we have now in our eye, and which has elicited these remarks. We hope, for the sake of the fair fame of our medical literature, that such a practice may never be repeated. *Verbum sat.*

The treatise of Mons. Velpeau needs no commendation from us. It will always be able to speak for itself. As a work of learning, it is without a rival in any language. The only approach to it, is the Treatise on Operative Surgery of Ernst Blasius, the distinguished Professor of Halle; a second edition of which was published in 1840. It was rather unfortunate that Velpeau's work fell into the hands of Dr. Townsend, who, as a translator, has certainly not done it justice. We do not think that we have ever seen a worse specimen of "French done into English," in any department of science or literature; and yet we would not speak irreverently of a man who has performed so great a service for his profession.

Dr. Townsend had the merit of an honest, if not a successful versionist; he had a great affection for his author, and his zeal to interpret him correctly is apparent in every line and paragraph of his translation. But he, nevertheless, often failed, chiefly because he had an imperfect knowledge of the French language, and an inadequate idea of the past and present state of operative surgery. His expressions are frequently most ludicrous; and his style rarely displays any of the graces of correct scholarship. He often retains French words and phrases whose meaning and import are at once palpable to the humblest intellect. It would have been well if the present editor could have revised Dr. Townsend's literary labors; but we can readily imagine that this would have been a task little short of that incident to supplying an entirely new translation. As a literary performance, therefore, nothing can be said in favor of the American edition of the work. The bad English which pervades the translation is equally conspicuous in the contributions of Dr. Mott. They all bear intrinsic evidence of hasty composition, circumlocution, inelegance, and even wretched grammar. The matter, fortunately, is good, but it is frequently so much buried in verbiage as to render it difficult of access. Besides, it is often very defective in its arrangement and collocation. Dr. Mott was evidently never intended for a great writer; and perhaps it was well that nature withheld from him so rare a gift, otherwise he might have been too vain of his accomplishments. Bonaparte wrote bad French, but he was a great chieftain, an acute statesman, and a most sagacious legislator. If Dr. Mott is not a great scholar, he is a great surgeon, whose deeds will convey his name securely down to the remotest ages of the world. God is chary of the favors he bestows upon mortals. He rarely concentrates in the same individual all the great qualities of human nature. John Hunter, with all his zeal, and his unrivalled powers as an interpreter of Nature's laws of healthy and diseased action, was a poor writer; so that now, after the lapse of nearly three-quarters of a century since his death, men experience much difficulty in unravelling the meaning of some of his harsh and ill-constructed sentences. But we are not on this account the less grateful for what this illustrious man has done for us, and for the same reason we should be thankful for the services of Dr. Townsend and Dr. Mott.

We regret that Dr. Mott's name has ever been associated with Velpeau's work. We are sure that it has never stood in need of his services. A plain, simple, faithful translation would have been quite sufficient to have secured for it a favorable reception from the profession of this country.

Velpeau needs no such endorsation, nor does Dr. Valentine Mott require such a parasitic connection to procure him readers of anything he may be inclined to publish. The Nestor of American surgery has reached his threescore years and ten, and it is therefore high time that he should furnish us, in a more enduring form than he has yet done, a record of his vast experience. A good book has said, whatever thou doest, do it quickly, and with all thy might; and we hope that Mons. Velpeau's annotator will profit by the injunction. He owes the profession a great original work on surgery.

We are gratified that the present edition has been brought out under the auspices of so able and distinguished a writer and surgeon as Dr. Blackman. We know of no gentleman in the profession in this country who would have executed the task with more success, in better taste, or in a more scholarly manner. Dr. Blackman wields a ready pen, and whatever he touches he adorns. His thorough acquaintance with operative surgery, his large personal experience in this branch of medicine, and his unwearied industry in the collection of facts, have admirably fitted him for bringing out an improved edition of this great work. His contributions comprise a large amount of matter in the form of notes, interpolations, and an appendix, affording thus a brief but accurate digest of the additions that have been made to operative surgery since the publication of the first edition, twelve years ago. Our only regret is that the matter in the appendix was not incorporated in the text, as this would have greatly facilitated reference. A good index is also still a desideratum, which we hope to see supplied in another edition.

